# Autofluorescence lifetime augmented reality as a means for real-time robotic surgery guidance in human patients

**DOI:** 10.1038/s41598-018-37237-8

**Published:** 2019-02-04

**Authors:** D. Gorpas, J. Phipps, J. Bec, D. Ma, S. Dochow, D. Yankelevich, J. Sorger, J. Popp, A. Bewley, R. Gandour-Edwards, L. Marcu, D. G. Farwell

**Affiliations:** 1 Department of Biomedical Engineering, University of California Davis, Davis, CA USA; 2 Institute of Physical Chemistry and Abbe Center of Photonics, Friedrich-Schiller University Jena, Jena, Germany; 3 Leibniz Institute of Photonic Technology e.V., Jena, Germany; 4 Department of Electrical and Computer Engineering, University of California Davis, Davis, CA USA; 5 Intuitive Surgical, Sunnyvale, CA USA; 6 Department of Otolaryngology-Head and Neck Surgery, University of California Davis, Sacramento, CA USA; 7 Department of Pathology and Laboratory Medicine, University of California Davis, Sacramento, CA USA; 8 Present Address: Institute of Biological and Medical Imaging, Helmholtz Zentrum München, Neuherberg, Germany; 9 Present Address: Jena Optronik, Jena, Germany

## Abstract

Due to loss of tactile feedback the assessment of tumor margins during robotic surgery is based only on visual inspection, which is neither significantly sensitive nor specific. Here we demonstrate time-resolved fluorescence spectroscopy (TRFS) as a novel technique to complement the visual inspection of oral cancers during transoral robotic surgery (TORS) in real-time and without the need for exogenous contrast agents. TRFS enables identification of cancerous tissue by its distinct autofluorescence signature that is associated with the alteration of tissue structure and biochemical profile. A prototype TRFS instrument was integrated synergistically with the da Vinci Surgical robot and the combined system was validated in swine and human patients. Label-free and real-time assessment and visualization of tissue biochemical features during robotic surgery procedure, as demonstrated here, not only has the potential to improve the intraoperative decision making during TORS but also other robotic procedures without modification of conventional clinical protocols.

## Introduction

Robotic surgery is becoming a preferred means of treatment for numerous cancers and diseases in areas such as head and neck, urologic, gynecologic, and thoracic surgery. This technology can provide high surgical precision and improve recovery times for the patient^1^. To date, most robotic interventions are performed with the da Vinci Surgical System^2,3^.

Irrespective of the surgical approach, the success of surgery relies on the complete excision of the diseased tissues. In most cancers, including head and neck cancer, surgery is often the first choice of treatment, depending on the prognostic factors of the patient, the tumor classification, and the potential morbidity associated with acquiring complete resection with negative surgical margins^4,5^. Frozen section biopsy is the most commonly employed technique for rapid margin assessment. Positive margins with disease present at the periphery of the resected specimen are associated with worse disease specific survival in many tumors^6,7^. Preventing positive margins is a key surgical objective and is now recognized as a quality metric for surgeons in many fields. While standard pre-operative imaging can provide tumor location, surgeons still rely on visual and/or tactile guidance to determine the interface between normal and abnormal tissue and histology to confirm microscopic clearance of abnormal disease. In robotic surgery^3,8^ the lack of tactile feedback limits the surgeon to visual assessment through the robot camera, thus increasing the possibility of positive margins and residual diseased tissue.

To minimize such risk, the da Vinci Surgical System employs advanced technologies including highly-magnified 3D vision and precisely controlled instruments^3^. The most recent integration of the FireFly module into the da Vinci Surgical System has resulted in a paradigm shift in surgical navigation. This near-infrared fluorescence (NIRF) imaging technology allows for the visualization of blood flow and related tissue perfusion employing indocyanine green (ICG) as the fluorescent agent. Recent studies have employed this technology for numerous fluorescence-guided robot-assisted interventions including, but not limited to, robotic oncologic surgery, lymph nodes identification, vascularization and organ and tissue perfusion imaging^9,10^.

Nevertheless, the fluorescence-guided surgery, primarily with ICG, encompasses two significant limitations. First, the field of view augmented with ICG fluorescence information provides a qualitative rather than quantitative assessment of the tumor delineation. ICG imaging is based on perfusion and accumulation in tissue due to leaky capillaries seen in many diseased states such as neoplasms. However, it is not a molecularly targeted imaging probe and thus lacks specificity for early neoplastic lesions or field cancerization^11,12^. Second, ICG fluorescence imaging requires the lights of the operating room (OR) to be turned off, with only a few recent studies trying to address this limitation^13^.

A different approach that has the potential to guide surgical interventions is based on tissue autofluorescence. This technique has demonstrated potential to provide diagnostic contrast for diverse cancers, including head and neck cancer^14,15^. Steady-state (intensity or spectral) autofluorescence techniques have been reported as potential diagnostic tools for oral cancer. Such measurements, however, are affected by the experimental conditions, including changes in excitation-collection coupling due to tissue motion, non-uniform illumination or fluorescence collection due to surface geometry, and photobleaching^16^. In contrast, time-resolved autofluorescence techniques can address these limitations by resolving the dynamics of the fluorescence decay (lifetime)^17–19^. Additionally, fluorescence lifetime provides a contrast mechanism that can be employed for the discrimination of endogenous fluorophores with overlapping spectra. By exploiting alterations in tissue structural and metabolic characteristics resulting from cancer autofluorescence, lifetime imaging has the potential to provide information about tissue molecular composition including enzyme cofactors involved in cellular metabolism, matrix proteins and inflammatory activity^20,21^. Recent studies demonstrate the ability of this approach to delineate head and neck tumors from surrounding normal tissues in patients^14,15^.

Current study demonstrates the first-in-human proof-of-principle for integration of an autofluorescence technique with a surgical robot and real-time augmentation of the surgical field of view with parameters encoding tissue diagnostic information for surgical guidance. Specifically we show (1) the synergetic integration of a scanning multispectral time-resolved fluorescence spectroscopy (ms-TRFS) system^22,23^ into the da Vinci Surgical System that enable real-time continuous evaluation of tissue biochemical features; (2) the evaluation of the functionality of the integrated system *in vivo* in swine models; and (3) the ability of the integrated ms-TRFS system to operate in human subjects undergoing TORS procedures and to provide real-time diagnostic contrast based on intrinsic tissue autofluorescence properties. While TORS is not as common as other applications of robotic surgery, there are many advantages of using it as a model to demonstrate the feasibility of integrating the ms-TRFS system into the da Vinci Surgical System workflow. The unique attributes of TORS, working with multitude of tissue types and providing robotic access to challenging anatomic locations, demonstrate the advantages of the robotic approach and the utility of the ms-TRFS system to query tissue composition and surgical margins during surgery. The integrated system is designed to collect and analyze ms-TRFS data in real-time from areas of interest in the oropharynx prior to and after surgical excision of cancer. The system also extracts fluorescence parameters related to tissue biochemical properties that are used to augment the robot’s field of view. In addition, current approach can serve as a framework for the clinical translation of other point-scanning optical techniques, such as elastic-scattering spectroscopy, diffused reflectance spectroscopy, or Raman spectroscopy^24–26^, to name a few.

## Results

### Integration of ms-TRFS and da Vinci Surgical Systems

**Figure 1:**
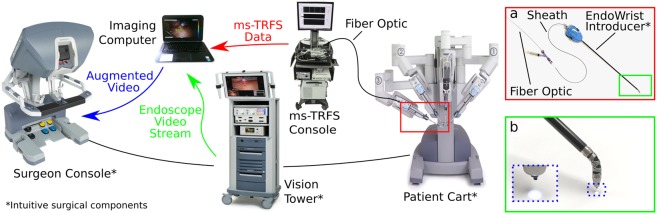
Integration of the ms-TRFS system into the da Vinci Surgical System. The da Vinci Surgical System is composed of three modules: the surgeon console that enables remote control of the robotic system, the patient cart where the surgical tools are mounted, and the vision tower used as an interface for the endoscopic camera. All components required to perform ms-TRFS measurements are packaged into a compact console. ms-TRFS measurements are performed using a sterile probe consisting of a single multimode fiber, integrated into an Intuitive Surgical tool (EndoWrist 5Fr Introducer). Tile (**a**) depicts the fiber inserting the sheath and both inserting the introducer. The distal end of the fiber exiting the introducer is shown in tile (**b**) and with higher magnification in the inset. The imaging computer receives both ms-TRFS data as well as a video stream from the endoscope so that the location of ms-TRFS measurements within the operating field is known. An overlay of the ms-TRFS data onto the video of the surgical field is then sent to the surgeon console for display. All images from the da Vinci Surgical System were reproduced from Intuitive Surgical with written permission.

The ms-TRFS system used for this study is packaged in a compact cart that allows minimal interference with OR procedures and is integrated into the da Vinci Surgical System (see Fig. 1 and Methods). The system can provide spectroscopic images registered with the operating field by combining freehand scanning of the optical fiber-probe with the white-light video stream acquired by the endoscopic camera (see Methods).

**Figure 2:**
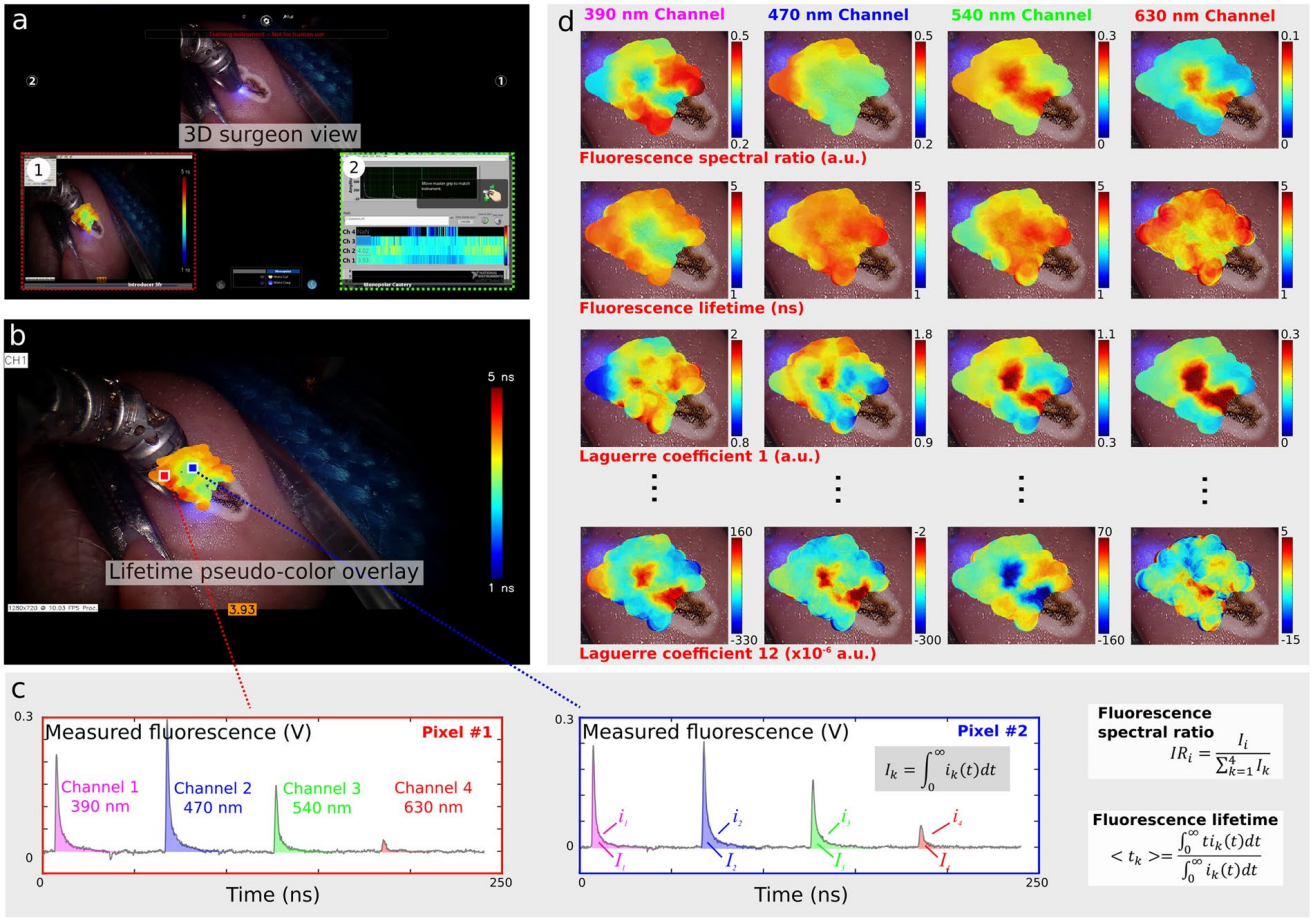
Augmentation of ms-TRFS derived data on the Surgeon console during oral cavity surgery. (**a**) The conventional three-dimensional visualization from the endoscope. The 445 nm aiming beam used to identify the location probed by ms-TRFS is visible at the distal end of the tool. The panels labeled with (1) and (2) correspond to the lifetime maps that can be visualized and the linear representation of lifetime values, respectively. (**b**) The white-light image augmented with lifetime values from channel 1 of the instrument. (**c**) The transient signals over the four channels of the system for two locations (pixels) measured. The fluorescence pulse transients from all four channels are simultaneously acquired and analyzed to all relevant emission parameters (i.e. integrated intensity, lifetime, and 12 Laguerre coefficients). (**d**) Matrix of the distribution maps for the autofluorescence parameters. Note, each of these maps can be displayed/augmented in real-time during the scanning procedure if needed.

Delivery of an additional visible aiming beam through the optical probe, as described in earlier work from our group^23^, enables dynamic tracking of the location probed by ms-TRFS. An illustration of how the information acquired by a point-measurement technique is transformed into a two-dimensional image with “memory” capability, resulting in detailed visualization of the fluorescence lifetime contrast, is displayed in Fig. 2. The two distinct formats to display the ms-TRFS derived parameters on the surgeon console (the two panels labeled with (1) and (2) in Fig. 2a) were demonstrated *in vivo* in porcine animal models. Supplementary Video 1 displays a scanning sequence inside the oral cavity from the animal’s tongue. For each location of the probe, it takes ~0.04 sec (practically instantaneously) to acquire, process/analyze and display the ms-TRFS parameters on the surgeons’ console. The augmented frame of Fig. 2b showcases the ability of the ms-TRFS system to display the tissue autofluorescence features on the da Vinci surgeon console once integrated into the da Vinci Surgical System. The endogenous fluorescence contrast originates from the different tissue constituents emitting fluorescence under the same excitation. Figure 2c depicts the transient signals acquired simultaneously for the four spectral bands of the system (see Methods) from two distinct areas (pixels) in the oral cavity. The wealth of fluorescence parameters retrieved from each fluorescence pulse transient during the scanning procedure is depicted in Fig. 2d. This includes the spectral ratio (i.e. intensity of one channel over the sum of intensities from all channels), lifetime, and the 12 Laguerre coefficients^27^.

Efficient and safe imaging in animal models and patients was achieved with several additional functionalities described in the following section. This includes means to account for the wide variations of signal observed during freehand scanning, the potential fouling of the probe with biological matter during imaging, and the limitation of tissue exposure to UV light during ms-TRFS measurements.

### Dynamic gain control

**Figure 3:**
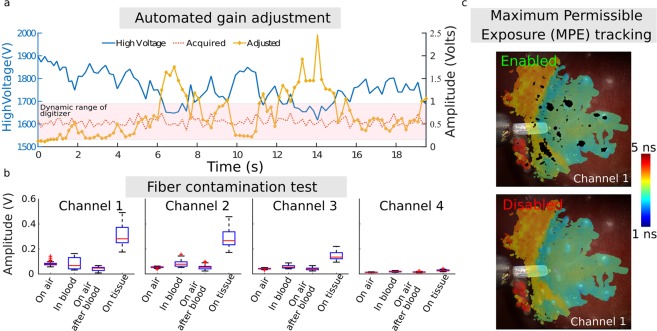
Safe and robust ms-TRFS data acquisition *in vivo* through the implementation of specialized functions. (**a**) The signal acquired from the photodetector (red dotted line) was within the dynamic range of the digitizer (red shaded area). This was achieved by modulating the bias voltage of the photomultiplier tube (blue line). The effective signal intensity can be recovered by combining the acquired signal with the bias voltage value (orange line). The data shown above (500 points) were obtained during *in vivo* measurements in the oral cavity of a swine. (**b**) The amplitude of four different conditions of measurement and for all four channels of the ms-TRFS system show that the fiber is not contaminated after immersion in blood. (**c**) The area of the field of view exposed to laser light is tracked during imaging and exposure is computed. This demonstration in swine displays areas where exposure is close to the limit as defined per ANSI Z1.36.1 in black to provide feedback to the surgeon about risk of overexposure (top). The lifetime maps are nevertheless constructed and can be assessed by disabling this feature (bottom).

Acquisition of robust ms-TRFS data during surgery depends on the system’s ability to account for sudden variations in fluorescence intensity. Such differences are due to variations in the fluorescence signal generated by different types of tissues encountered during surgery and changes in excitation-collection geometry caused by variations in the probe-to-tissue distance when performing freehand scanning. The fluorescence signal generated by tissue is collected by the optical probe, spectrally resolved and sent to a photodetector (microchannel plate photomultiplier tube) where it is converted to an electrical signal, amplified and sampled by a high-speed digitizer (see Methods). High signal-to-noise ratio ms-TRFS data are obtained for the highest signal voltage that can be generated by the photodetector and amplifier without causing saturation of the digitizer. This constraint can be reconciled with the wide variations of collected fluorescence signal noted above by modulating in real-time the bias voltage - and hence gain - of the photodetector, based on the intensity of the measured signal. This high dynamic range measurement scheme based on closed loop control of the detector gain is adapted from a method reported by our group^22^. Figure 3a depicts representative results from the evaluation of this method during an *in vivo* scan and demonstrates its ability to compensate for rapid changes in acquired intensity. The scanned area consists of 500 consecutive points and includes normal mucosa, taste buds, and charred tissue. The amplitude of the acquired transient signals over the 500 points considered is depicted in Fig. 3a (red dotted line), along with the applied gain (blue solid line). As seen, the gain varies between ~1700 Volts and ~1900 Volts, whereas the amplitude remains relatively constant and always within the digitizer’s dynamic range (i.e. 0.05 and 0.8 Volts, red transparent region). Despite the difference in tissue types and probe position, the SNR for all 500 points remained stable (48.2 ± 2.4 dB) throughout the scan, validating the robustness of the dynamic gain control. This is further confirmed by the relatively constant amplitude of the acquired transient signals (0.54 ± 0.1 V). The variation of fluorescence signal intensity is recovered by correcting the measured signal (red dotted line) with the applied high voltage bias (blue line) and the gain/bias voltage characteristic of the photomultiplier tube (0.03 V^−1^) and shows the signal that would be obtained without adaptive gain control (solid orange line). We observe that without the adaptive gain control this signal would fall outside the dynamic range of the system for a relatively large number of measured points.

### Optical fiber probe contamination (blood, cauterization byproducts) and cleaning strategy

Besides the system’s ability to account for rapid changes in fluorescence signal, acquisition of robust ms-TRFS data also relies on the ability to keep the distal end of the fiber clean throughout the entire intraoperative procedure. Fiberoptic contamination due to adhesion of either blood or surgical debris (i.e. cautery byproducts) may affect the characteristics of the measured fluorescence. The optical probe distal end was treated with a hydrophobic coating (see Methods). The ability of this coating to limit contamination of the probe following contact with blood was evaluated *in vivo* in the oral cavity of a swine. The ms-TRFS measurements were performed in 4 configurations: clean fiber reference measurement (n = 54 measurements), in contact with blood (n = 204 measurements), in air following contact with blood (n = 207 measurements) and close from tissue (n = 75 measurements). The signal collected in each configuration for all four channels of the instrument is displayed in Fig. 3b. Imaging in blood led to a small increase in detected signal due to the underlying tissue, with no significant difference in the signal before and after exposure to blood. Channel 1 shows a minor increase in the intensity before exposure to blood, compared to the one after, which is due to possible proximity to tissue with high collagen or elastin content (i.e. although in air the fiber is still inside the oral cavity). Additionally, strong signal could be acquired from tissue following exposure of the optical probe to blood, thus demonstrating the efficacy of the hydrophobic layer at the fiber’s distal end in preventing blood contamination.

Cauterization byproducts can also contaminate the fiber resulting in unwanted increase of fluorescence background. A sequence showing the ability to clean the fiber in the oral cavity using saline is demonstrated in Supplementary Video 2. A clean fiber placed inside the oral cavity of the swine was situated a few centimeters away of any tissue before cauterization, after cauterization, and after cleaning using a saline solution. The smoke and projections from cauterization resulted in a fluorescence signal that can be observed in all 4 ms-TRFS detection channels. Saline flushing removed such fluorescent debris. The cleaning process requires only a few seconds and does not affect the standard surgical procedure or present any risk for the patient (Supplementary Video 2).

### Tracking of Maximum Permissible Exposure (MPE)

Exposure of human tissue to laser light must be performed in accordance with the American National Standard for Safe Use of Lasers (ANSI Z136.1-2014, Laser Institute of America)^28^. Based on light pulse characteristics, an MPE value is determined to prevent thermal damage to tissue (see Methods). The exposed locations of the tissue are identified to create the ms-TRFS augmented video streams and a cumulative exposure for each pixel of the field of view is determined. When the cumulative exposure reaches 50% of the permissible exposure, the corresponding pixels are marked in black to warn the surgeon that such areas should not be further exposed (Fig. 3c).

### Integrated ms-TRFS and da Vinci Surgical Systems: *In vivo* evaluation in human patients (case study)

**Figure 4:**
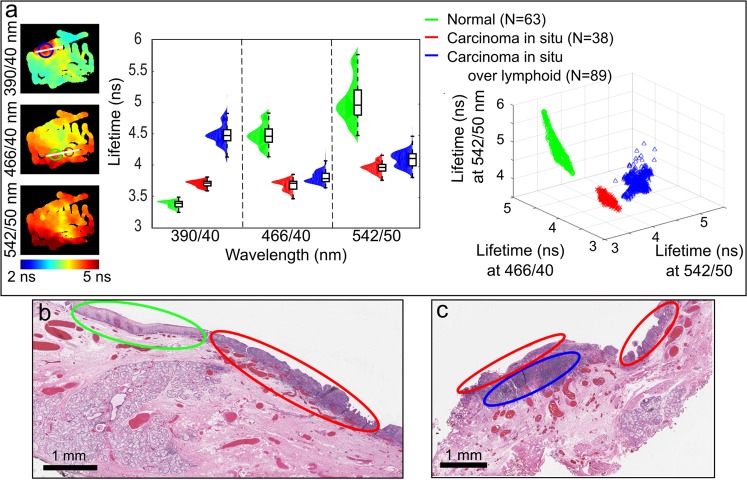
Discrimination of different tissue types through measurements with the ms-TRFS system integrated into the da Vinci Surgical System. (**a**) Lifetime maps from the first three channels of the ms-TRFS system with three regions of interest highlighted: carcinoma *in situ* (red circle), carcinoma *in situ* over lymphoid tissue (blue circle), and normal tissue (green circle). The distribution of lifetime values over the three spectral channels (middle) demonstrates the contrast between these regions. Three clusters can be seen in the three-dimensional scatter plot of the lifetime values (right). (**b**) A histology section demonstrating normal (green ellipse) versus carcinoma *in situ* (red ellipse) tissues. The depicted section aligns with the line crossing the green and red circle of panel (a). (**c**) A histology section demonstrating carcinoma *in situ* (red ellipse) versus carcinoma *in situ* over lymphoid (blue ellipse). The depicted section aligns to the line crossing the blue circle of panel (a).

The ability of the system to allow the surgeon to acquire and display ms-TRFS measurements during a TORS procedure was evaluated in four patients – case study. Figure 4 shows the results from ms-TRFS interrogation of surgical margins prior to tumor resection in one of the patients. The measurements, as captured from the surgeon’s console, that produced the results shown in Fig. 4 can be seen in Supplementary Video 3. Results from the other three patients can be seen in Supplementary Data 2 and Supplementary Fig. 2.

The data collected from this patient demonstrate the potential of ms-TRFS measurements to provide diagnostic information and delineate tumor from normal tissue during TORS procedures. The results are summarized in Fig. 4a. Specifically, three regions of interest were identified by the pathologist and co-registered with the lifetime maps constructed during the scanning procedures: carcinoma *in situ*, carcinoma *in situ* over normal lymphoid tissue, and normal tissues, (Fig. 4a, left). The lifetime distribution of these areas (Fig. 4a, middle) reveals that the carcinoma *in situ* over normal lymphoid tissue presents higher lifetime values than the other two tissue types in the 390/40 nm channel of the ms-TRFS system. The normal tissue, on the other hand, presents significantly higher lifetimes with respect to the carcinoma *in situ* in the other two spectral channels. The contrast between the three tissue types and the intrapatient variability is further demonstrated in the scatter plot of Fig. 4a (right), where the lifetime values are plotted for all three channels. Two representative histologic sections used to identify these three regions are shown in Fig. 4b,c.

## Discussion

Here we present the first integration of a multispectral label-free fluorescence lifetime technique into the da Vinci Surgical System and demonstrate the ability of this technique to (1) record robust time-resolved fluorescence data during TORS procedures in human patients, (2) augment autofluorescence lifetime data onto conventional white-light images seen on the da Vinci surgeon console in real-time, and (3) provide contrast between distinct tissue types, including alteration by surgical procedures in human patients. Although TORS presents numerous benefits for the patient, it removes tactile guidance leaving only visual inspection for the assessment of tumor margins. The technique reported here can provide a novel surgical guidance technique with potential to become a valuable tool during robotic surgical procedures.

We showed that the ms-TRFS technique can be integrated into the da Vinci Surgical System without affecting the standard functions of either system. The fiberoptic probe can be easily coupled with the da Vinci 5Fr Introducer; ms-TRFS data acquisition and visualization is implemented with the standard video output connections, already available in the robotic system. After the ms-TRFS system preparation, the ms-TRFS measurement is controlled through a single foot-switch, which is placed at the surgeon console and directly accessed by the surgeon. Since the ms-TRFS data visualization is in real-time, the surgeon can dynamically assess the signal quality and interact accordingly (i.e. reduce the probe-surface distance or reduce/increase the scanning speed). Also, by recording each pixel’s exposure, the MPE can be approximated and the surgeon can visually be notified to avoid the regions where the MPE was reached. Future plans include the disabling of the excitation source in case these regions are visited even though the MPE threshold has been reached. This measure, in combination with the conservative definition of the applied MPE threshold will ensure that the true MPE will never be reached during a scanning session. Visualization of the acquired data is implemented through the robot’s TilePro function; thus, it does not interfere with the standard robotic platform currently used in clinical practice. None of the two surgeons participating in the study presented herein had any issue or difficulty in performing the measurements, even though one of them had no prior experience with the system (see Methods and Supplementary Data 2), which further increases its prospect for clinical translation. Moreover, the same approach can be adapted to other non-TORS procedures conducted with the robot (Supplementary Fig. 1 and Supplementary Data 1).

One important aspect of the technology described herein is its ability to acquire robust autofluorescence data from tissue. In part this is due to the high dynamic range of the ms-TRFS system, which is enabled by the dynamic control of the gain applied to the detector. This feature ensures that data are acquired regardless of rapid changes in quantum efficiency or light collection geometry. In addition, the saline flushing method developed enables the rapid cleaning of the distal end of the fiber from cauterization byproducts or smoke. Finally, the hydrophobic coating of the fiber’s distal end prevents blood contamination, minimizing signal attenuation risks.

The developed approach for intraoperative guidance presents one basic limitation challenge; the tissue motion impact on acquired data. While tissue motion can be controlled with instrumentation in TORS, this may not be as possible during laparoscopic procedures. This challenge can be addressed reasonably well with the development of methods that could detect the motion and/or tissue deformation and correspondingly transform the lifetime maps. Moreover, during intraoperative guidance, surgeons rely on the assessment of relatively small areas, close to the interrogated locations. As demonstrated by this study, the technique described herein can image such areas without being degraded from motion blurring.

Earlier fluorescence lifetime instrumentation based on fiberoptic probes with direct access to distinct areas in the oral cavity has demonstrated potential to differentiate between cancerous and normal tissue in the oral cavity^14,15,29^. However, these techniques were not able to either provide real-time feedback on tissue biochemical features or to augment the fluorescence parameters on tissue surface. They lack the “memory” function or the ability to trace-back the evaluated areas. This function is important for intraoperative guidance, as well as for further validation with standard histopathology analysis. In this work, we demonstrate for the first time a technique able to provide continuous real-time visualization of areas interrogated by a fiber optic probe, streamed to the surgeon console and to generate diagnostic contrast based on tissue autofluorescence properties.

While the scope of this study was primarily to demonstrate the integration of ms-TRFS system into the da Vinci Surgical System and its functionality during conventional TORS procedures, we have also shown the potential of the ms-TRFS data to discriminate different tissue types and/or conditions, namely carcinoma *in situ*, carcinoma *in situ* over lymphoid tissue such as tonsils, and normal tissue. Importantly this was achieved based on tissue intrinsic fluorescence properties without the need of contrast agents. These findings are anticipated to steer future studies to quantify the discriminatory power of ms-TRFS.

In addition, the technique demonstrated here could be adapted to other point-scanning tissue diagnostic modalities as it can overcome the lack-of-memory limitation without the need of specialized equipment or fixed geometry requirements^26^. Moreover, the methodology presented herein can easily be modified to allow for integration of any fluorescence spectral band of interests and display of measured quantities derived from light-tissue (e.g. elastic scattering spectroscopy, diffuse reflectance spectroscopy, or Raman spectroscopy).

## Methods

### Study design

The objective of this study was to determine whether ms-TRFS, a point-scanning technique, could be employed during TORS for real-time assessment of tissue biochemical characteristics, including delineation of tumor. To test this approach, we integrated the ms-TRFS system in the da Vinci Surgical System and performed a series of *in vivo* measurements, both in animal models and human patients undergoing TORS. The measurements were targeted to validate the performance of ms-TRFS to acquire and visualize data online without interfering with the standard clinical practice during TORS.

### Animal models

The functionality of the integrated ms-TRFS system into the da Vinci Surgical System was evaluated *in vivo* on three swine (N = 3). Animal studies were approved by the Intuitive Surgical Institutional Animal Care and Use Committee (protocol #009, entitled Imaging and Visualization in Surgery). All methods, facilities and transportation complied with current legal guidelines and regulations. Anesthesia was used for all measurements.

These experiments were designed to (a) investigate the capability of the ms-TRFS system to operate in an *in vivo* setting comparable with that used in humans, (b) optimize ms-TRFS measurements and data acquisition protocols, (c) evaluate the ability of the ms-TRFS system to collect data from different tissue types inside the oral cavity, (d) evaluate conditions (blood, surgical debris, cautery-derived effects) that might affect the ms-TRFS signals and to engineer solutions to overcome such challenges. In addition, the prospect of employing this system during laparoscopic interventions was investigated.

All measurements were implemented inside the oral cavity of the animal models, except from the laparoscopic measurement. For this, two small incisions in the abdominal wall of the animal allowed the EndoWrist Introducer and the endoscope to access the area of interest. The surgeon performing the interventions identified specific areas of interest and performed the scanning measurements with the da Vinci Surgical System. As depicted in the results and videos, there was no specific scanning pattern. Instead the surgeon could freely scan the region of interest, as long as this motion was not obscuring the field of view of the endoscope.

### Human patients

The ability of the ms-TRFS system to operate synergistically with the da Vinci Surgical System was tested in four human patients (N = 4) during standard TORS interventions. The study was performed with approval from the University of California, Davis Institutional Review Board and in accordance with all relevant guidelines. All patients provided written informed consent. This study was designed to determine whether ms-TRFS could be applied intraoperatively to provide continuous feedback about tissue biochemical features, as well as to augment the conventional endoscopic white-light images. Two surgeons participated in the study. One surgeon also performed the swine procedures, while the other had no prior experience with the ms-TRFS system (see Supplementary Fig. 2).

Before the robotic surgery the endoscope and the EndoWrist instrument with the fiber from the ms-TRFS system were inserted inside the oral cavity of the patient under anesthesia. The surgeons identified the region of interest, based on pre-operative planning, and lifetime measurements were acquired by scanning over that region. As in the animal models, surgeon-directed scanning pattern with the EndoWrist instrument was easily accomplished with fluorescent analysis being performed in real time. For all patients, scanning time was less than 5 minutes without any significant delays or interruptions to the standard clinical procedures. Results of this study are presented here and to the authors’ knowledge this is the first-in-human application of intraoperative fluorescence lifetime imaging during robot-assisted interventions.

### Integration of ms-TRFS into the da Vinci Surgical System

The instrumental apparatus employed in this study consisted of a prototype scanning ms-TRFS system interfaced with the da Vinci Si Surgical System via a 5Fr EndoWrist Introducer (Fig. 1).

#### Optical Instrumentation

In brief, the optical instrumentation consists of a wavelength selection module (WSM) composed of dichroic and band-pass filters that simultaneously spectrally-resolve the fluorescence emission in four spectral channels: 390/40 nm (Channel 1), 466/40 nm (Channel 2), 542/50 nm (Channel 3), and 629/53 nm (Channel 4)^30^. A continuous-wave solid-state laser (445 nm, TECBL-50G-440-USB, WORLD STAR TECH, Toronto, ON, Canada) coupled into Channel 2 of the WSM allows for injection of an aiming beam via the same path as the fluorescence excitation beam^23^. The incident power of the aiming beam is approximately 3 mW. The four channels are connected to optical fibers of distinct lengths (acting as delay channels) coupled at the distal ends into a single microchannel plate photomultiplier tube (MCP-PMT, R3809U-50, 45 ps FWHM, Hamamatsu, Japan). After being detected by the MCP-PMT and boosted by an RF amplifier (AM-1607-3000, 3 GHz bandwidth, MITEQ, USA), the fluorescence signals are temporally resolved by a high sampling frequency digitizer (12.5 GS/s, 3 GHz, 8-bit, 512 Mbytes, PXIe-5185, National Instruments, Austin, TX, USA) at 80 ps time intervals. Auto-fluorescence signal induced by the aiming beam (continuous-wave) is filtered-out by the AC-coupled amplifier. A micro Q-switched laser frequency tripled to 355 nm with a repetition rate equal to 2 KHz (Teem Photonics, France) is used for fluorescence excitation. To enable easy transportation from/to the operating room the system is integrated in a movable cart (Fig. 1).

#### da Vinci Surgical System

The da Vinci Surgical System (Intuitive Surgical, Sunnyvale, CA, USA) is a teleoperated surgical system for minimally invasive operations. It consists of three major parts: (i) the surgeon console, through which the surgeon performs the operation while viewing a high resolution three-dimensional image of the interrogated tissues; (ii) the patient cart, which is docked to the patient during surgery and has four robotic arms; and (iii) the vision tower which is equipped with a high-definition, three-dimensional endoscope and image processing equipment that provides images of the patient’s anatomy in real-time. The system received its first clearance from the FDA in 2000. In 2008 the system received clearance to perform TORS for selected malignant lesions of the pharynx and larynx. During this study, the ms-TRFS system was integrated into the da Vinci Si Surgical System (Fig. 1), but other models are also suitable.

#### Hardware Integration

A customized fiberoptic probe enabled the integration of the ms-TRFS system into the da Vinci Surgical System. A single multimode 400 μm core diameter silica fiber (BFH48-400, Thorlabs Inc., Newton, NJ, USA) was introduced to the working channel of the 5FR EndoWrist Introducer and used for both fluorescence excitation and collection. This introducer consists of a wrist with three degrees of freedom that allows aiming of the fiberoptic probe for any tissue orientation. The wrist has a tight bend (~12 mm radius), thus fatigue testing was performed to evaluate whether the imaging fiber can withstand the strain. Although no fiber damage was noticed, a >25 mm radius was maintained during scanning sessions to match the fiber’s short-term bend radius (22 mm). In addition, the employed fibers are compatible with low temperature gas plasma sterilization, while the tough tefzel fiber buffer allowed for several sterilization cycles (i.e. use of the same fiber for multiple patients) without any fiber damage. A sterile sheath (4F IMPRESS Vertebral, Merit Medical Systems, Inc. Salt Lake City, UT, USA) was used to protect the fiberoptic probe during measurements.

Preliminary testing showed that the fiberoptic distal end is prone to contamination due to adhesion of either blood or surgical debris (i.e. cautery byproducts). To address this, the fiber was treated with a hydrophobic coating (Aculon, San Diego, CA, USA) and a syringe port for a water flush was added to the sheath. In the presence of hydrophobic coating, simple retraction of the fiber into the sheath and low volume flush is sufficient to clean the fiber tip.

To streamline the flushing process and better protect the fiber outside of the imaging sequence, a motorized telescope functionality was integrated into the system. A module that clips to the sheath and locks onto the fiber with a hemostat (Fig. 1) was developed. This enables retraction of the fiber within the sheath and exposure only during the imaging process. All elements of the mechanism are either sterilizable or protected within a sterile sheath (i.e. the stepper motor).

#### Software integration

The visualization schemes developed (i.e. the ms-TRFS interface shown in Fig. 2c and the augmented white-light images of Fig. 2b) are shared with the surgeon console through the TilePro interface and thus provide the surgeon with real-time biochemical and/or functional information by means of fluorescence decay dynamics.

To integrate the framework described in^23^ into the da Vinci Surgical System, a frame-grabber (Epiphan Systems Inc., Ottawa, ON, Canada) was connected between the vision tower and the imaging laptop. The transmitted frames (i.e. one of the two channels of the endoscope) are captured and processed in parallel with the fluorescence transient signals as described in^23^. Finally, the resulting images are displayed on the surgeon console through the TilePro interface.

### Data acquisition, processing, and visualization

#### Online data acquisition and processing

Two features were adopted to optimize the data acquisition during TORS procedures. The first consists of a method enabling optimization of the signal amplitude against the dynamic range of the digitizer. A closed-loop control algorithm reported earlier^22^ provides feedback to the high voltage power supply. The latter determines the gain of photon multiplication and alters the signal amplitude dynamically during data acquisition. The second consists of remote laser triggering and data acquisition by the surgeon from the surgeon console via a foot pedal. This feature not only gives control of the system to the surgeon but also prevents unintended exposure from the excitation light.

The data processing module is incorporated into the system’s software platform using LabVIEW (National Instruments, Austin, TX, USA) and implemented in the embedded controller of the digitizer. The approximation of the lifetime values from the acquired transient signals was implemented through a non- parametric non-negative least-squares deconvolution with Laguerre expansion. This method has been described in depth by our group elsewhere^27^ and represents the basis for achieving real-time quantification of lifetime values.

#### Real-time data visualization

When the aiming beam module is enabled, the acquired transient signals are transmitted to an external laptop. The deconvolution algorithm (implemented in the laptop) is applied in parallel to the image processing. The framework of employing an aiming beam to augment white-light images with quantitative information (biochemical, functional, statistics outcomes, etc.) has been described by our group recently^23^. In addition to visualizing in real-time the lifetime values from the four channels of the WSM, the software also monitors the compliance to MPE as defined by the American Standard for Safe Use of Lasers ANSI Z136.1^28^. Specifically, MPE is determined based on wavelength, energy, pulse duration and repetition rate of the UV and aiming beams. Cumulative exposure of the illuminated areas, detected from the endoscope image, is determined under the conservative assumption that the fluence on tissue corresponds to the fiber being in contact with tissue. When the cumulative exposure reaches a set fraction of the permissible exposure (50%), the corresponding pixels are marked in black to provide feedback to the surgeon that those locations should not be exposed further.

## Supplementary information

Supplementary Video 1

Supplementary Video 2

Supplementary Video 3

Supplementary Information

## Data Availability

All data are available by the corresponding authors under reasonable request.
