# Supplementary material for: Autofluorescence lifetime augmented reality as a means for real-time robotic surgery guidance in human patients
Source: Sci Rep. 2019 Feb 4;9:1187. doi: 10.1038/s41598-018-37237-8 (PMC6362025; doi:10.1038/s41598-018-37237-8)
Supplement: Supplementary file 4 — Supplementary Information [file 41598_2018_37237_MOESM4_ESM.doc]

**Autofluorescence lifetime augmented reality as a means for real-time robotic surgery guidance in human patients**

D. Gorpas, J. Phipps, J. Bec, D. Ma, S. Dochow, D. Yankelevich, J. Sorger, J. Popp, A. Bewley, R. Gandour-Edwards, L. Marcu, D. G. Farwell.

**Supplementary Data 1**:

**Application of the ms-TRFS system in laparoscopic procedures with the da Vinci Surgical System.**

The potential of the ms-TRFS to be employed in robotic surgical procedures other than TORS, in particular laparoscopic procedures in the abdominal cavity, is depicted in Supplementary Fig. 1. In contrast to intervention in the oral cavity, where motion is typically not present, the organs in abdominal cavity are characterized by some field motion. Although the ms-TRFS technique was found sensitive to blurring due to motion when relatively large areas are measured, it can provide consistent lifetime estimation for smaller regions of interest because of its relatively high frame rate.

Supplementary Fig. 1a shows a standard white-light frame captured from the surgeon console while performing scanning ms-TRFS measurements inside the abdominal cavity. As in the oral cavity, visualization of quantified lifetime values can be enabled either as augmented with lifetime values white-light frames (Supplementary Fig. 1b for Channel 1) or as a live stream of color-coded values (Supplementary Fig. 1c). The interrogated organs within this experiment were the gallbladder, the liver, and the stomach of an *in vivo* swine. During these measurements ms-TRFS data successfully visualized lifetime contrast between each organ. Specifically, for Channel 1, the lifetime values were 4.80±0.10 ns (n=426) for gallbladder, 4.33±0.20 ns (n=447) for liver, and 4.65±0.13 ns (n=280) for stomach and statistical analysis via one-way ANOVA test indicates statistically significant differentiation between these lifetime values (p«0.001). The color-coded linear display of the lifetime values also depicts contrasts between the different organs; however, the lack of the augmented information makes the online identification of the contrast’s sources rather challenging. Lifetime contrast is further detected at an organ level, as seen in Supplementary Fig. 1d, e for the lifetime values from Channel 1 on the large intestine.


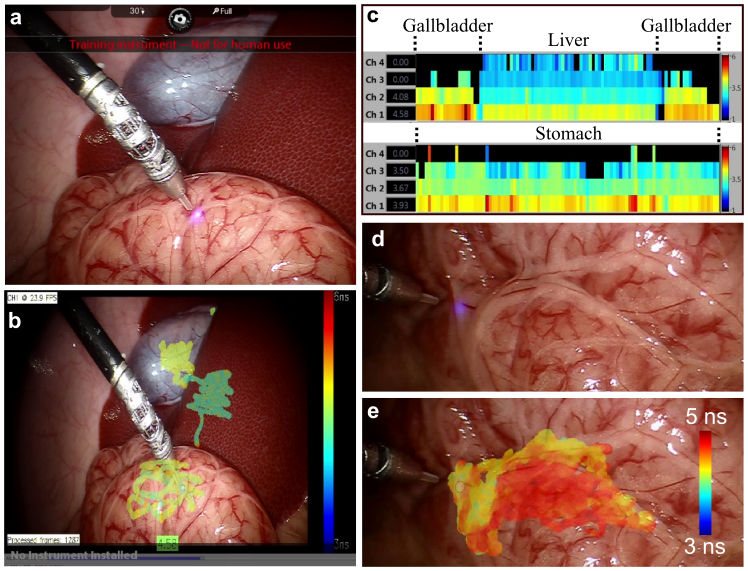


**Supplementary Figure 1 |** **Endoscopic application of the ms-TRFS system in the abdominal cavity of a swine.** (**a)** White-light frame as captured by the endoscope. **(b)** The same frame augmented with lifetime values from Channel 1 with the fiber targeting the gallbladder, the liver, and the large intestine, correspondingly. Lifetime values between the three organs measured present significant differentiation (p«0.001). **(c)** Lifetime can also be visualized as color-coded stream. Lack of “memory” makes it difficult to translate these measurements back to the white-light frames. **(d)** A white-light frame and **(e)**, the corresponding augmented frame of the large intestine. Lifetime contrast from Channel 1 is visible between septation (connective tissue) and mesenteric tissue (fatty).

**Supplementary Data 2**:

**Integrated ms-TRFS and da Vinci Surgical Systems: *In vivo* evaluation in human patients (case study) – Results from additional patients**

Supplementary Fig. 2 shows results acquired by additional patients. Specifically, Supplementary Fig. 2a depicts characteristic screenshots from the surgeon’s console during the data acquisition from the first patient. The first two panels (from the left) correspond to measurements prior the tissue excision, while the other two after the excision. This imaging session targeted to the validation of the hardware integration, the ms-TRFS system operation assessment inside the operation theatre, and the training of the surgeon and other clinical personnel. Nevertheless, the integration was so straight-forward that there was no impact at all on the standard clinical procedures.

Supplementary Fig. 2b showcases the tests performed with the imaging ms-TRFS module enabled. These measurements were acquired from a second patient and by a surgeon without prior experience either with the hardware system or the software. Nevertheless, application of the technology described herein was seamless and the surgeon managed to successfully measure the region of interest.

The results from a third patient are shown in Supplementary Fig. 2c and 2d. Specifically, a screenshot of the surgeon’s console during a scanning procedure is shown in Supplementary Fig. 2c, with the default three-dimensional visualization of the scene on top and the augmented with lifetime values from Channel 2 of the ms-TRFS system on the bottom. The lifetime values, as well as the intensity ratios, from all four channels of the system are shown in Supplementary Fig. 2d. During these measurements, the functionality of the MPE monitoring was also assessed and in the augmented with lifetime values frame from the first two channels this feature was enabled and is the black area in Supplementary Fig. 2d. As described in Methods, MPE monitoring is rather conservative (by assuming the fiber being in contact with the tissue) and thus black regions are informative and do not represent any risk to the patient.


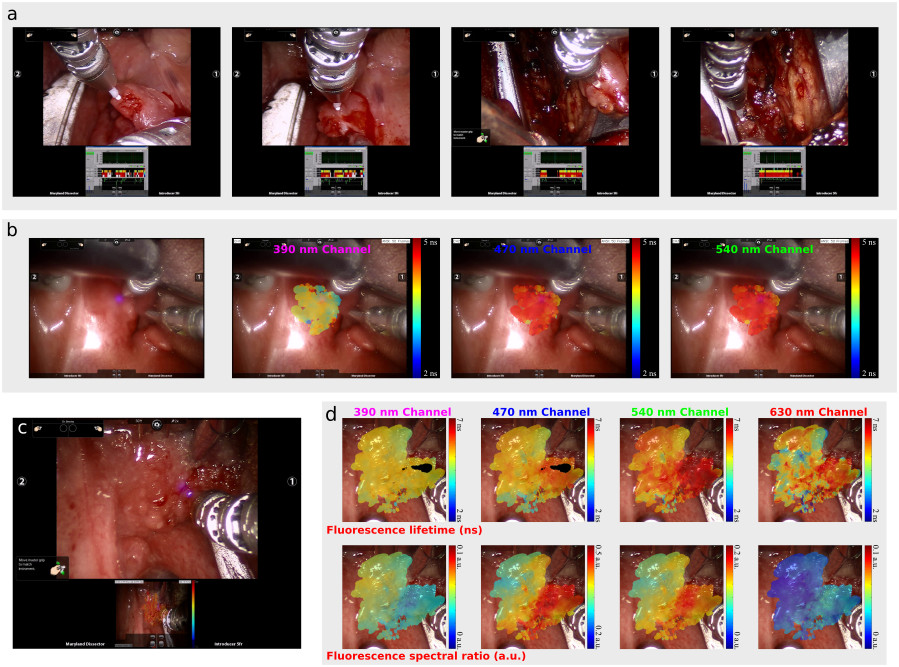


**Supplementary Figure 2 |** **Validation of synergetic ms-TRFS and da Vinci Surgical system in human patients.** **(a)** Assessment of the ms-TRFS system integration with the da Vinci Surgical System in human patient in the OR for the first time. Multiple areas inside the oral cavity were measured to fully validate the seamless integration. **(b)** Assessment of the performance of the lifetime imaging module. These data were acquired by a surgeon without any prior experience with either the hardware or the software components of the ms-TRFS system. **(c)** A screenshot of the surgeon’s console during measurements on a third patient. **(d)** Fluorescence lifetime values and intensity ratios from all four channels of the ms-TRFS system are estimated in real-time and can be projected onto the white-light frames acquired by the da Vinci endoscope. In the lifetime maps from the first two channels of the ms-TRFS system the MPE monitoring was enabled and areas exceeding the estimated threshold are painted black.

**Supplementary Videos**

**Supplementary Video 1. Scanning sequence in the oral cavity: *In vivo* swine.**

Real-time in vivo fluorescence lifetime measurements in the oral cavity of a swine with the combined ms-TRFS and da Vinci Surgical System. Movie captured from the surgeon console demonstrating ms-TRFS measurements from tongue with superficial cauterization. In addition, the movie demonstrates the feasibility to switch rapidly the display to show the emission in distinct fluorescence channels without pausing or stopping the scanning process. The top panel corresponds to the endoscope output of the da Vinci Surgical System. The bottom left panel corresponds to the ms-TRFS GUI which is not activated and only displays the raw transient signals. The bottom right panel shows the endoscope output augmented with lifetime data.

**Supplementary Video 2. Fiber cleaning through saline flashing: Demonstration in the oral cavity of *in vivo* swine**

The sequence depicts: 1) Clean fiber positioned in the proximity to the cauterization site. 2) Byproducts of the cauterization contaminate the fiber distal end, which results in residual fluorescence signal. 3) This residual signal removed by flashing the fiber with saline, thus without removing the fiber from the oral cavity. The movie was captured from the surgeon console. Top panel corresponds to the conventional endoscope output, while bottom panel depicts the ms-TRFS GUI. Through the GUI the operator can assess the contamination of the fiber by observing the raw transient signals, or the generated lifetime values. Ideally, when the fiber is clean the signals amplitude should be approximately zero (i.e. at the noise level of the system) and no lifetime values should be quantified.

**Supplementary Video 3. TORS procedure in human patient with integrated real-time visualization (augmentation) of lifetime information**

The top panel depicts the conventional endoscope output, the bottom left panel depicts the white-frame images augmented with lifetime values, and the bottom right depicts the ms-TRFS GUI populated only with the raw transient signals. The movie was captured from the surgeon console. The contrast in lifetime values from Channel 1 are due to carcinoma in situ, carcinoma in situ over normal lymphoid, and normal tissue that were confirmed by histology (see Fig. 4 of the manuscript). The movie’s last few frames depict the MPE (visual feedback) as observed at the location of the fiber tip.
